# Chebulagic acid from *Terminalia chebula* causes G1 arrest, inhibits NFκB and induces apoptosis in retinoblastoma cells

**DOI:** 10.1186/1472-6882-14-319

**Published:** 2014-08-29

**Authors:** Naresh Kumar, Gangappa D, Geetika Gupta, Roy Karnati

**Affiliations:** School of Life Sciences, University of Hyderabad, Gachibowli, Hyderabad 500046 India

**Keywords:** Chebulagic acid, Retinoblastoma, Apoptosis

## Abstract

**Background:**

Plants are the valuable source of natural products with important medicinal properties. Most of the approved anti cancer drugs have a natural product origin or are natural products. Retinoblastoma is the most common ocular cancer of children. Although chemotherapy is the preferred mode of therapy, a successful treatment for retinoblastoma requires enucleation. Chebulagic acid (CA) from *Terminalia chebula* was shown to have anti-proliferative properties in the studies on cancerous cell lines. Due to anti cancer properties of CA and due to limitation in treatment options for retinoblastoma, the present study is undertaken to understand the role of CA on the proliferation of retinoblastoma cells.

**Methods:**

Anti proliferative potential of CA was determined by MTT assay. The expression levels of various cell death mediators in retinoblastoma cells with CA treatment were assessed by Western blotting. Flowcytometer analysis was used to estimate the mitochondrial membrane potential (MMP) and to determine the percentage of cells undergoing apoptosis.

**Results:**

The present study showed CA inhibited the proliferation of retinoblastoma cells in a dose dependent manner. CA modulated MMP, induced release of Cytochrome *c*, activated caspase 3 and shifted the ratio of BAX and Bcl2 towards cell death. G1 arrest, noticed in CA treated cells, is mediated by the increase in the expression of CDK inhibitor p27. CA treatment also decreased the levels of NFκB in the nucleus. This decrease is mediated by suppression in degradation of IκBα.

**Conclusion:**

CA has shown significant anti proliferative potential on retinoblastoma cells. Our findings clearly demonstrate that CA induces G1 arrest, inhibits NFκB and induces apoptosis of retinoblastoma cells.

## Background

Natural products from plants are considered as a valuable source of active drug substances and a majority of modern drugs. Drugs derived from plants or microbes which are in preclinical development or in clinical phase are discussed in detail elsewhere [1]. In addition to the stand alone natural products, there is also a growing interest in the combination of natural products, being effective or efficacious in a particular pathological condition and such combinations were also approved for human use by regulatory authorities [2]. Plant derived natural products were also explored from treatment of cancers. Paclitaxel, camptothecin, etoposide, vinblastine and vincristine are all plant derived natural products approved for clinical use in many cancers. Epidemiological studies also show the beneficial effects of some fruit and vegetable diet against cancers [3, 4]. Bioflavonoids, isoflavonoids, β carotenes, phenols, polyphenols, catechins, alkoloids, lectins were shown to have anti proliferative potential in several cancers [5–9].

Natural products alone or in combination with standard drugs were shown to be effective against retinoblastoma [10–12]. Retinoblastoma is the most common ocular tumor of children. In the majority of cases the survival rate in retinoblastoma is almost 90% but successful treatment of retinoblastoma is achieved by enucleation [13]. At present treatment options include radiotherapy, chemotherapy, thermotherapy and brachytherapy [13, 14]. Many complications arise due to the conventional therapies such as impaired vision due to cataracts and retinopathy [13], gastrointestinal disorders, neurotoxicity [15–19]. Carboplatin, etoposide and vincristine are synergistically used in chemotherapy of retinoblastoma [13]. Secondary cancers are induced due to the conventional therapies in children of lower age [14, 20–22].

Inhibition of the molecular pathways having role in cell proliferation is a widely used strategy in anti cancer drug development. NFκB is an anti apoptotic transcription factor which plays an important role in the cell survival signaling. Interaction with IκB makes NFκB to reside in the cytoplasm as a dimer. Cell proliferation inducers mediate rapid degradation of IκB, promotes translocation of NFκB to the nucleus and induces the expression of several anti apoptotic protein including BCL2 family members [23, 24]. p27 regulates cell cycle transition and increase in the expression of p27 arrests the cells in G1 phase and restricts entry into S phase. p27 is phosphorylated by CDK 2–cyclin E and this phosphorylation of p27 results in its degradation through ubiquitin mediated proteosomal degradation [25]. NFκB activity is constitutively required for the survival and proliferation of retinoblastoma cells [26].

Natural products are being explored for effective therapies and to reduce complications due to conventional chemotherapy in retinoblastoma. Few studies were successful in showing anti proliferative potential. Resveratrol inhibits proliferation of retinoblastoma cells by activating mitochondrial mediated apoptosis [27]. Abelmoschus moschatus extracts showed potent anti oxidant and anti proliferative activity in retinoblastoma [10]. Plant derived natural products like peruvoside, ouabain, neriifolin, digoxin, and digoxigenin induced greater than 75% inhibition in at least one retinoblastoma cell line [11]. Paclitaxel (PTX), a diterpene taxane, induced apoptosis in retinoblastoma cells along with beta lactophone [12].

In our previous studies we have shown CA from *Terminalia chebula* as a potent suppressor of lipopolysaccharide induced inflammation in mouse macrophages [28]. CA further showed broad spectrum anticancer effects on HCT-15 (colon), COLO-205 (colon) cell lines [29]. *Terminalia chebula, a* member of the *Combretaceae* family, native of India, is being used in alternative medicine. The dried fruits are being used for treatment for conditions of asthma, cough, bloody stools, heart and bladder disease [30]. The fruits are rich in high molecular weight tannins [31, 32]. Benzopyran tannins are one of the major components in the fruits of *Terminalia chebula.* CA, a benzopyran tannin, was reported as a COX-2/5-LOX dual inhibitor [29]. CA has been shown to inhibit ROS generation [33] and anti- hyperglycemic activity [34]. CA was also reported to alleviate arthritis in mice models [35] and inhibited LPS-induced Nitric oxide [36]. CA and punicalagin were shown to inhibit HSV-1 entry in A549 human lung cells by preventing binding, penetration, and cell-to-cell spread [37]. In addition to these reported studies, CA is the main constituent of Triphala, a well known ayurvedic medicine used to treat allergies and common health disorders in India.

Due to several important medicinal properties of CA and limitation of the current conventional therapies in retinoblastoma, the present study is undertaken to understand the effect of CA on the proliferation of retinoblastoma cells and elucidate the molecular mechanisms involved.

## Methods

### Chemicals

DMEM, FBS, Rhodamine 123, Propidium iodide were purchased from Gibco BRL. MTT [3-(4,5-dimethylthiazol-2-yl)-2,5-diphenyl-2*H*-tetrazolium bromide], protease inhibitor cocktail and TMB/H_2_O_2_, DAPI, Ac-DEVD-pNA, z-VAD-FMK were from Sigma-Aldrich. Nitrocellulose and PVDF membranes were from Millipore. Monoclonal antibodies of Cytochrome *c*, BAX, Bcl2, p27, NFκB-p65, IκBα and β-actin were from Cell Signaling technologies and Millipore. All the other chemicals and reagents were purchased from local companies and are of molecular biology grade. Chebulagic acid was isolated from fruits of *Terminalia chebula* as described previously [29]. Fruit material of *Terminalia chebula* (Combretaceae) authenticated by Prof. K. Seshagirirao, and the dried drupes were deposited at University of Hyderabad Herbarium (UH) [University of Hyderabad, Hyderabad 500046, India] repository with Specimen No. 1006-KRRMR.

### Cell culture

Retinoblastoma cells Y79 were grown in Dulbecco’s modified Eagle’s medium (DMEM) supplemented with 10% (v/v) heat-inactivated FBS, 100 IU/ml penicillin, 100 mg/ml streptomycin and 2 mM L-glutamine. Human corneal epithelial cells were grown in MEM alpha medium supplemented with EGF (0.1 mg/l) and insulin (5 mg/l). Both cultures were maintained in a humidified atmosphere with 5% CO_2_ at 37°C. The cultured cells were passed twice each week, seeding at a density of approx. 2 × 10^3^ cells/ml. Cell viability was determined by the Trypan Blue dye exclusion method before seeding for each experiment.

### Cell proliferation assay

Cell proliferation of Y79 cells with CA treatment was determined by the MTT assay. Y79 cells were seeded in 96-well plate in the presence or absence of CA (0.001, 0.01, 0.1, 0.5, 1, 5, 10, 25, 50 and 100 μM) for 24 h at a density of 5 × 10^3^ cells/well in a volume of 100 μl medium. After incubation, 20 μl of MTT at a concentration of 5 mg/ml was added. After 4 h incubation at 37°C, 100 μl of lysis buffer was added to each well. Plates were agitated for 1 min and absorbance was read at 570 nm on a multi-well plate reader. The percentage of the inhibition of proliferation was calculated as a fraction of control (without CA treatment). To assess the effect of CA on non cancerous cells, Human corneal epithelial cells were used under similar treatment conditions.

### Cell morphology analysis

Y79 cells (1 × 10^5^) were incubated with CA (50 μM) for 24 h. Cells were observed and photographed for morphological changes under a phase contrast inverted microscope.

### Nuclear morphology and DNA fragmentation analysis

Y 79 cells at a density of 1 × 10^5^ were grown overnight in a cell culture dish. The cells were then incubated with CA (50 μM) for 24 h. After incubation, cells were washed with 1 × PBS and mounted on to the slide with the mounting medium containing DAPI. The slides were then observed for changes in nuclear morphology in an Olympus inverted fluorescence microscope. For DNA fragmentation assay, Y 79 cells (5 × 10^6^ cells) were incubated at 37°C with CA (50 *μ*M) for 24 h. After treatment, cells were washed in cold PBS and lysed in a buffer containing 50 mM Tris (pH 8.0), 1 mM EDTA and 0.2% Triton X-100 for 20 min at 4°C. After centrifugation at 14 000 *g* for 15 min, the supernatant was treated with proteinase K (0.5 mg/ml) and 1% SDS for 1 h at 50°C. DNA was extracted twice with phenol and precipitated with 140 mM NaCl and 2 volumes of ethanol at −20°C overnight. DNA precipitates were washed twice in 70% (v/v) ethanol, dissolved in TE buffer, and treated for 1 h at 37°C with RNase A. Finally, DNA preparations were electrophoresed in 1% agarose gel, stained with ethidium bromide and observed under UV light.

### Cell cycle analysis by flow cytometer

Y 79 cells were seeded at a density of 4 × 10^5^ in 6-well culture plates, grown overnight in medium containing 10% FBS with or without CA (50 μM) for 24 h. After treatment cells were washed with 1 × PBS. For the cell cycle analysis, cells were fixed in 70% ice cold ethanol, washed with 1 × PBS, incubated with RNase A (0.1 mg/ml) and stained with propidium iodide (PI) (50 mg/ml). Flow cytometric analyses were performed by using a BD Calibur machine (San Jose, CA, U.S.A.) as previously described [38]. The fluorescence intensity of PI was detected with FL2-H detector. X axis on the histogram represents PI fluorescence intensity and Y axis represents number of cells at particular fluorescence intensity.

### Measurement of mitochondrial membrane potential (MMP)

Y79 cells, at a density of 4 × 10^5^ were seeded in 6-well culture plates and cultured with or without CA (50 μM) for 24 h. After the treatment, cells were incubated with Rhodamine 123 (10 μg/ml) for 30 min. After the incubation, cells were washed with 1 × PBS. MMP was assessed on a flow cytometer. Data were collected using the data acquisition program CELL Questpro and fluorescence was measured using an FL-1H detector as previously described [38]. Ten thousand cells were analysed per sample. The fluorescence intensity of Rhodamine was detected with FL1-H detector. X axis on the dot plot represents Rhodamine fluorescence intensity and Y axis represents SSC-H (side scatter) indicating the granular content of the cells.

### Assessment of caspase 3 activity

Caspase-3 activity in the lysates of Y79 cells treated with CA (50 μM) and untreated cells. 100 μg of total protein was incubated with Caspase 3 specific peptide (Ac-DEVD-pNA) at a final concentration of 200 μM at 37°C for 2 h in dark. The absorbance was read at 400 nm. Cells were pre-treated with a pan-caspase inhibitor (z-VAD-FMK) for 3 h at a concentration 20 μM before the cells were treated with CA (50 μM) and cell proliferation was estimated by MTT assay.

### Western blotting

Y79 cells were seeded at a density of 5 × 10^6^ in 100 mm culture dishes. They were incubated with CA (50 μM) for 0, 3, 6, 12, 24 and 48 h. After incubation with CA, cells were washed with 1 × PBS and suspended in cell lysis buffer with 1 mM PMSF, 10 μg/ml leupeptin, 20 μg/ml aprotinin and phosphatase inhibitor cocktail. After 30 min of intermittent vortexing at 4°C, the cell lysate was centrifuged (10 000 g) for 30 min, and the supernatant were used as the whole cell extract. Nuclear and cytoplasmic extracts were also prepared as described previously [39]. The protein content was estimated by the Bradford method [40]. 100 μg of either total or nuclear or cytosolic protein from each treatment were resolved on 7–12% SDS PAGE gels along with protein molecular weight markers and then transferred on to nitrocellulose or PVDF membranes. Membranes were stained with 0.5% ponceau stain to check the transfer. The membranes were blocked with 5% (w/v) BSA and then incubated with the primary antibodies [BAX (1:250 dilution), Bcl-2,(1:500 dilution), Cytochrome *c* (1:250 dilution), p27 (1:500 dilution), NFκB-p65 (1:500 dilution), IκBα (1:500 dilution), β-actin (1:1000 dilution)] in 10 ml of antibody-dilution buffer (1 × Tris-buffered saline and 0.05% Tween 20 with 1% BSA) with gentle shaking at 4°C for 8–12 h and then incubated with peroxidase or alkaline phosphatase conjugated secondary antibodies. The signals were detected by using peroxidase or alkaline phosphatase substrates. The band intensity was quantified by Image J software.

### Statistical analysis

Results are reported as the Mean ± S. E. M. for three independent experiments. Statistical analysis was carried out by Student’s t test. A p-value of less than 0.01 for each concentration versus control was considered to indicate significance.

## Results

### Y79 cell proliferation was inhibited by CA

**Figure 1:**
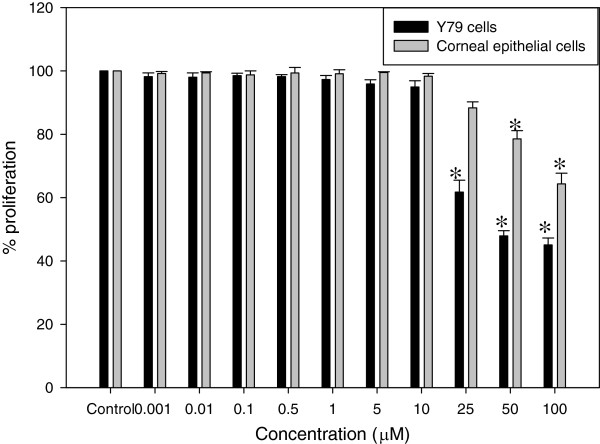
**Effect of CA on the proliferation of retinoblastoma and normal human corneal epithelial cells.** Y79 and HCE cells were treated with 0.001, 0.01, 0.1, 0.5, 1, 5, 10, 25, 50 and 100 μM concentration of CA for 24 h. The percentage of viable cells with treatment was calculated in comparison with untreated control cells. The number of cells in the control was taken as 100%. Data was represented as mean ± S.E.M of three independent experiments. *P < 0.01 vs untreated control was considered to indicate significance.

Y79 cells were treated with CA (0.001, 0.01, 0.1, 0.5, 1, 5, 10, 25, 50 and 100 μM) for 24 h and cell proliferation was determined by the MTT assay. CA treatment decreased the proliferation of Y79 cells in a dose dependent manner. At a concentration of 50 μM of CA, a 50% decrease in Y79 cell proliferation was observed (Figure 1) in 24 h. Normal human corneal epithelial (HCE) cells were treated with CA at same concentration as that of Y79 cell treatment. CA at 50 μM concentration could inhibit only 20% of HCE cell proliferation compared to 50% in Y79 cells. Further experiments were carried out with 50 μM CA as 50% inhibition of Y79 cell proliferation was observed at this concentration.

### Y79 cell morphology altered with CA treatment

**Figure 2:**
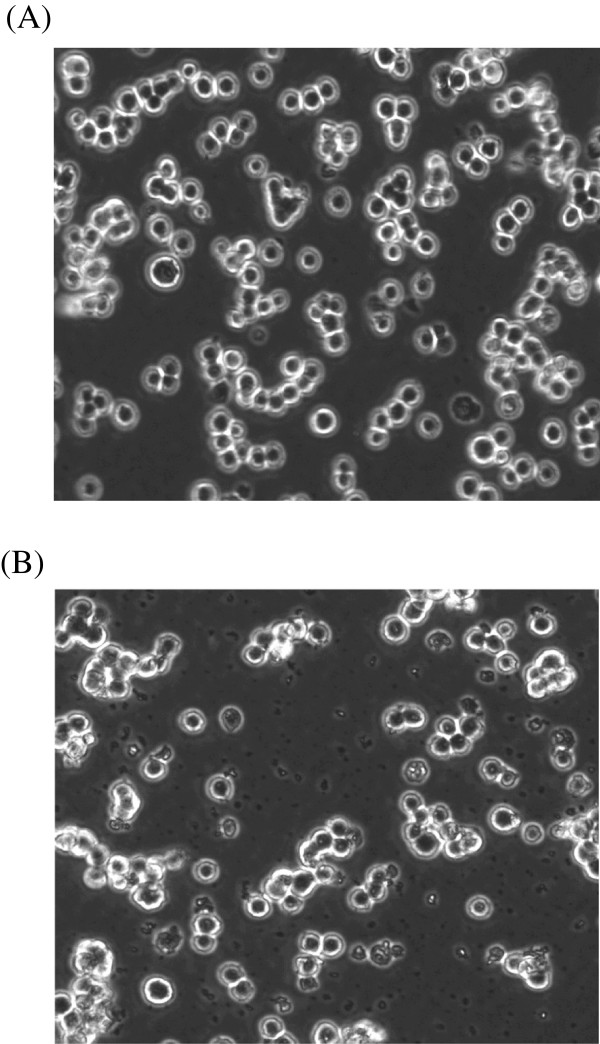
**Effect of CA on morphology of Y79 cells. (A)** Control cells **(B)** CA (50 μM; 24 h) treated cells.

Treatment of cells with cell death inducing agents alters the morphology of cells. Disruption of membrane and formation of membrane blebs are commonly observed. Untreated Y79 cells grow in grape like clusters (Figure 2A), but with CA treatment, morphology and growth pattern of the cells changed considerably. Y79 cells with CA treatment showed distorted morphology, membrane blebs and less clusters (Figure 2B).

### CA induced nuclear and DNA fragmentation in Y 79 cells

**Figure 3:**
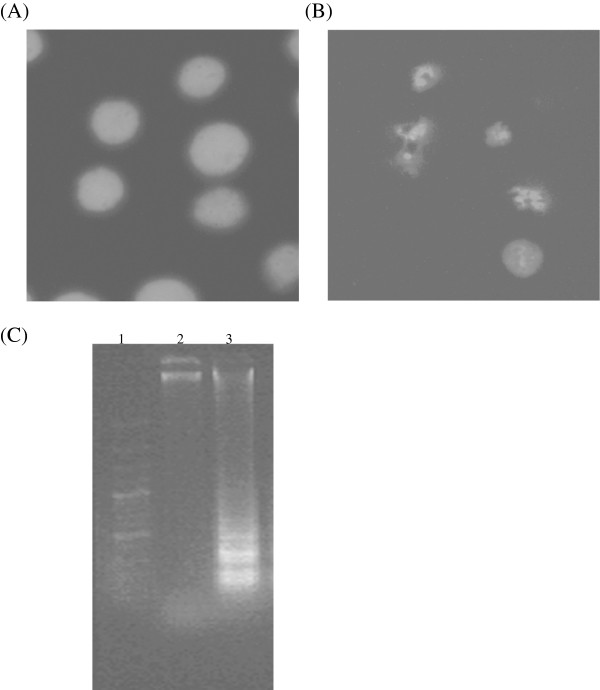
**Effect of CA on nucleus morphology and DNA fragmentation of Y79 cells. (A)** DAPI stained nucleus of control cells **(B)** DAPI stained nucleus of CA (50 μM; 24 h) treated cells **(C)** Lane 1, 100 bp DNA ladder; lane 2, DNA of control cells; lane 3, DNA of CA (50 μM; 24 h) treated cells.

Nuclear fragmentation, which is observed during apoptosis, is observed using a nuclear stain DAPI. In CA (50 μM) treated cells, fragmented nuclei were observed (Figure 3B) and the nuclei were intact in untreated control cells (Figure 3A). Cells treated with CA (50 μM) showed fragmentation of DNA, similar to the cells undergoing apoptosis (Figure 3C).

### Mitochondrial membrane depolarization was observed with CA treatment

**Figure 4:**
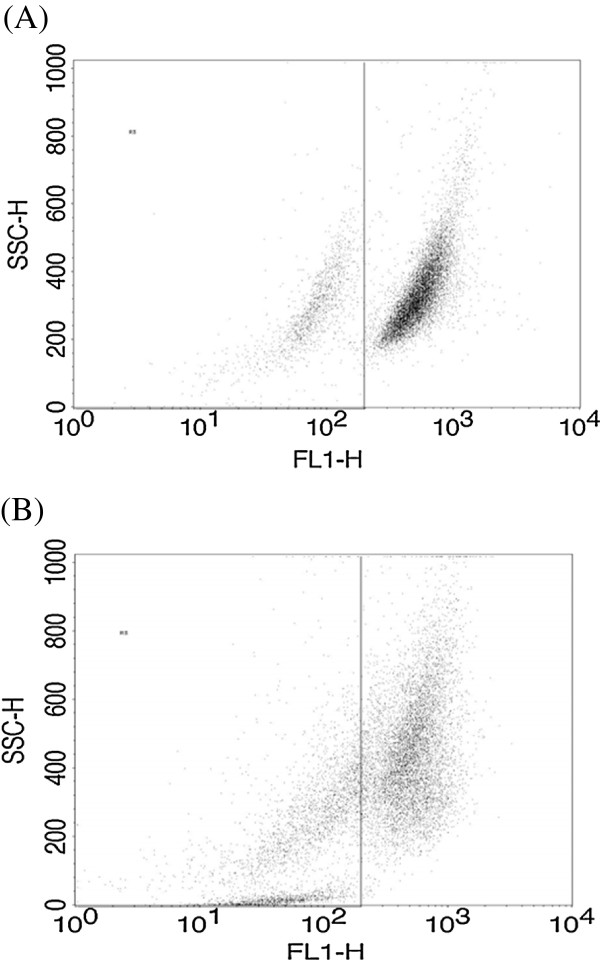
**Effect of CA on mitochondrial membrane potential of Y79 cells.** Cells were treated with CA (50 μM) for 24 h and incubated with Rhodamine 123 (10 μg/ml) for 30 min fluorescence was quantified by FACS. **(A)** Control cells **(B)** CA (50 μM; 24 h) treated cells.

Mitochondria play a key role in apoptosis. A change in the membrane potential of mitochondria is a critical step in the induction of apoptosis. MMP was assessed using Rhodamine 123 by flow cytometric analysis. In Figure 4, X represents FL1-H, detector for Rhodamine in the flow cytometer. Y represents SSC-H, detector for granularity of cells. The cells on the higher scale of FL1-H represent cells with higher membrane potential. In control untreated cells majority of cells are on higher FL1H scale (Figure 4A). Treatment with CA 50 μM, induced a shift in the significant cell population towards the lower scale of the FL1-H indicating depolarization of mitochondria (Figure 4B) and the treatment also resulted in decrease in cell granularity i.e. SSC-H, due to cell death.

### Ratio of Bcl2 and BAX shifted towards apoptosis with CA treatment

**Figure 5:**
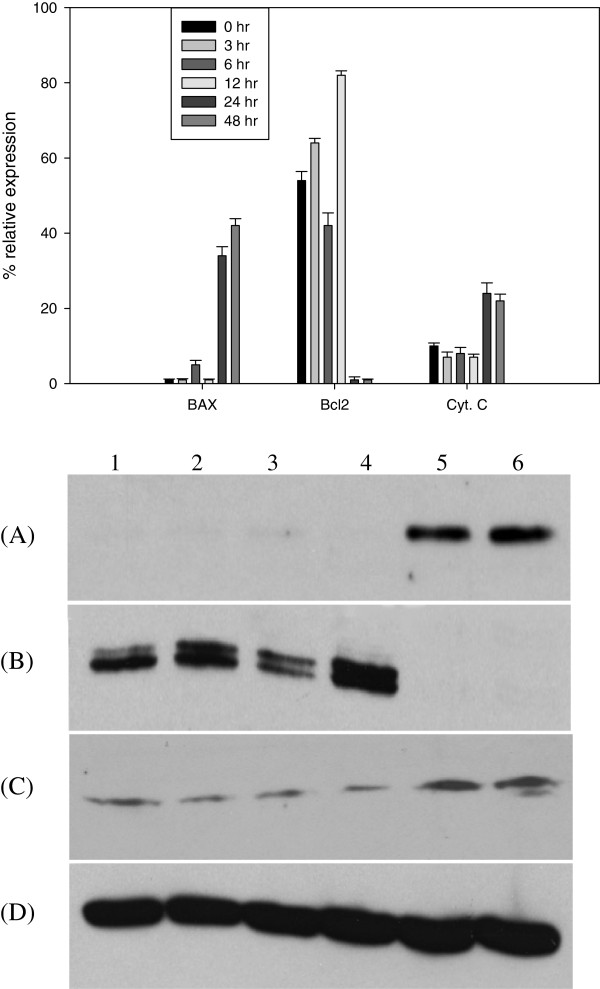
**Effect of CA on expression of cell death mediator proteins.** Y79 cells treated with CA (50 μM) for different time periods (0, 3, 6, 12, 24 and 48 h). Equal amounts of total protein (BAX, Bcl2 and β-actin) and cytosolic protein (Cytochrome *c)* (100 μg) was analysed by SDS- PAGE 7–12%, and after electrophoresis, proteins on the gel were transferred on to nitrocellulose membrane and probed with protein-specific antibody. Lane 1, 0 h; lane 2, 3 h; lane 3, 6 h; lane 4, 12 h; lane 5, 24 h; lane 6, 48 h. **(A)** BAX; **(B)** Bcl2; **(C)** Cytochrome *c* **(D)** β-actin. Bar graphs represents mean relative expression of protein normalised against β-actin.

Ratio of Bcl2 and BAX proteins decides the fate of the cells towards cell survival or cell death. Due to the decrease in the expression of anti-apoptotic protein Bcl2 and increase of expression of pro-apoptotic protein BAX, the cells die of apoptosis. Y79 cells treated with CA (50 μM) showed a time dependent increase in expression of BAX (Figure 5A) and decrease in expression of Bcl2 until 24 h and total absence at 48 and 72 h (Figure 5B).

### CA induced the release of Cytochrome *c*

Release of Cytochrome *c* into the cytosol as a result of mitochondrial depolarization initiates the intrinsic pathway of apoptosis. Western blot analysis with the cytoplasmic extracts devoid of mitochondria with anti-Cytochrome *c* antibodies was performed. A time dependent increase in Cytochrome *c* release in Y79 cells treated with CA (50 μM) was observed (Figure 5C). Untreated cells showed no release of Cytochrome *c*.

### CA induced apoptosis is Caspase 3 dependent

**Figure 6:**
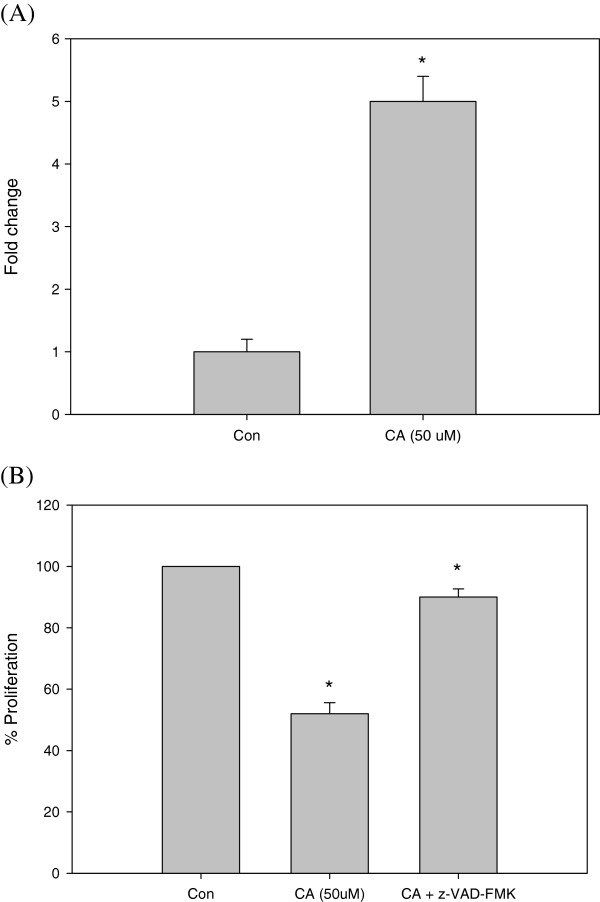
**Effect of CA on activation of caspase 3. (A)** Cells were treated with CA (50 μM) for 24 h, and lysates were analysed for caspase 3 activation. **(B)** Proliferation of CA (50 μM) treated Y79 cells for 24 h in the presence and absence of pan caspase inhibitor, z-VAD-FMK.

Caspase-3 activity increased in the lysates of Y79 cells treated with CA (50 μM) compared to that in untreated cells (Figure 6A). To show that CA induced cell death is caspase 3 dependent, MTT assay was done with CA, in the presence and absence of a pan-caspase inhibitor (z-VAD-FMK). Caspase inhibitor could inhibit the CA induced cell death in Y 79 cells (Figure 6B).

### CA treatment increased the number of Y79 cells in hypodiploid and G1 phases

**Figure 7:**
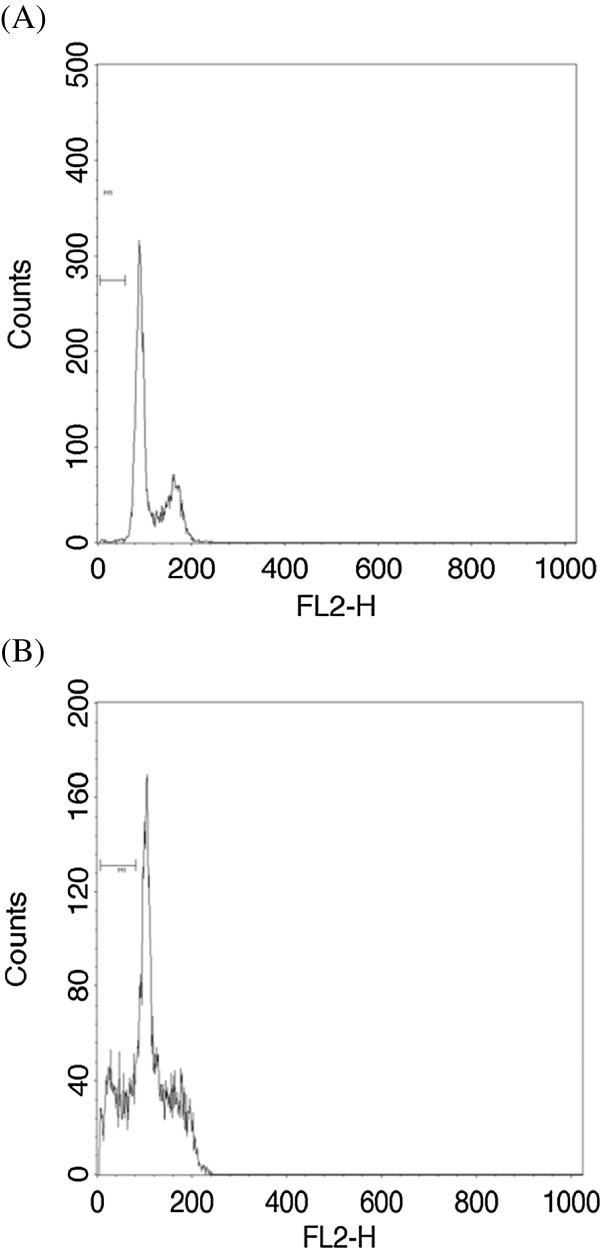
**Effect of CA on the cell cycle of Y79 cells.** Cells were treated with CA (50 μM) for 24 h, fixed, and stained with propidium iodide, and the DNA content was quantified by FACS. The number of cells in hypodiploid (subG0/G1) and G1 phase is expressed as a percentage of the total number of cells. **(A)** Control cells **(B)** CA (50 μM; 24 h) treated cells.

Number of hypodiploid cells increases with apoptosis. Induction of apoptosis with CA treatment was quantified by flowcytometric analysis. X represents FL2-H, detector for Propidium Iodide in the flow cytometer. Y represents counts, representing number of cells at particular FL2-H. Propidium Iodide is a DNA binding dye and proportion of fluorescence emission is based on quantity of DNA in the cell. In a dividing cells, the quantity of DNA in different phases of cell cycle varies as G1 < S < G2/M. The marker (M1) represents the cells before G1 peak, indicating the cells which are having less DNA (hypodiploid) than G1 phase cells. These hypodiploid cells are the cells undergoing cell death. In the Figure 6, we can clearly observe, the increase in number of cells before G1 peak with CA (50 μM) treatment compared to untreated cells. Untreated control cells showed prominent G1 (59%), S (12%) and G2/M (27%) phases with minimal 1% in sub G1 hypodiploid stage (Figure 7A). Treatment with CA increased the% of cells in sub G1 phase and G1 phase to 12% and 76% respectively (Figure 7B). These results indicate that CA treatment arrested the cells in G1 stage of cell cycle and also induced apoptosis.

### CA induces G1 arrest and inhibits NFκB

**Figure 8:**
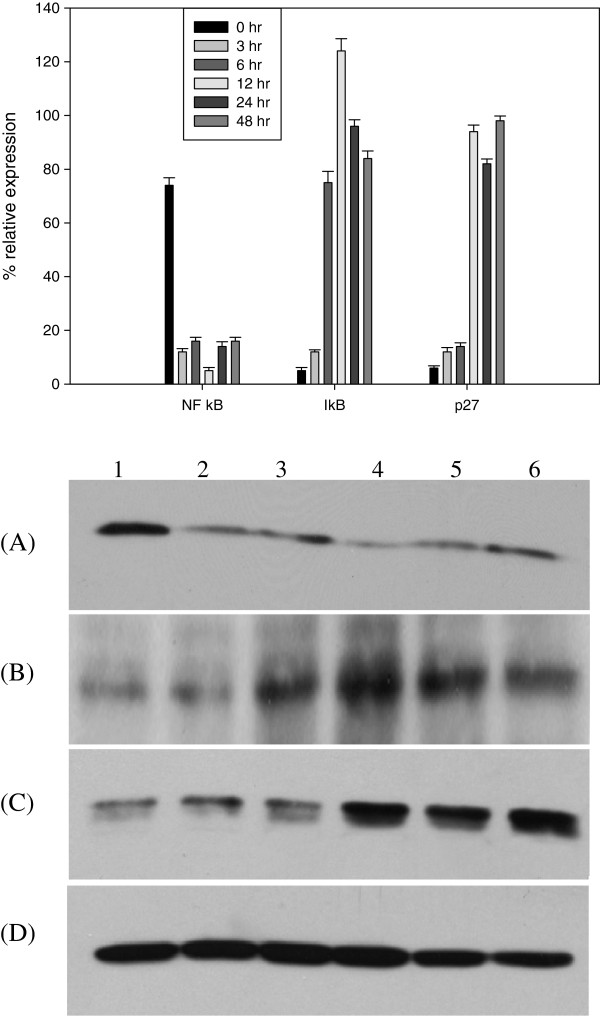
**Effect of CA on the expression of NFκB-p65, IκBα and p27 proteins.** Y79 cells treated with CA (50 μM) for different time periods (0, 3, 6, 12, 24 and 48 h). Equal amounts of total protein (IκBα, p27and β-actin) and nuclear protein (NFκB-p65) (100 μg) was analysed by SDS- PAGE 7–12%, and after electrophoresis, proteins on the gel were transferred on to nitrocellulose membrane and probed with NFκB-p65, IκBα, p27and β-actin specific antibodies. Lane 1, 0 h; lane 2, 3 h; lane 3, 6 h; lane 4, 12 h; lane 5, 24 h; lane 6, 48 h. Bar graphs represents mean relative expression of protein normalised against β-actin. **(A)** NFκB-p65, **(B)** IκBα, **(C)** p27, **(D)** β-actin.

p27 and NFκB are important regulators of cell cycle progression and proliferation. As increase in% of cells in G1 phase was observed, the expression levels of p27 were estimated. With CA (50 μM) treatment, a time dependent increase in expression of p27 was observed (Figure 8C), indicating CA mediated G1 arrest is through p27. Levels of NFκB are altered with proliferative status of cells. NFκB-p65 levels decreased in the nuclear extracts of Y79 cells with CA treatment (Figure 8A), where as IκBα levels increased (Figure 8B). These results indicate CA suppressed the degradation of IκBα, there by inhibiting the translocation of NFκB to nucleus.

## Discussion

Plants are the source of many clinically used potent anti-cancer molecules [1]. The enormous diversity of the plants was not explored and screened extensively for potential therapeutic molecules [41, 42]. Exploration of plants for natural products even makes it more relevant these days, with the advances in the methods and technologies to prepare analogs from the isolated natural products [43]. Our previous reports have shown the anti-cancer properties of natural products isolated from various sources [44–46]. Our group has shown CA isolated from *Terminalia chebula* inhibits LPS induced inflammation [28], inhibits proliferation of colon cancer cells [29]. In addition to these properties several groups have shown beneficial properties of CA [33–37]. Current therapies to retinoblastoma are limited necessitating the patient to undergo enucleation. Due to several medicinal properties of CA, the present study is planned to understand the effect of CA on retinoblastoma cell line, Y79, and to understand the molecular mechanisms involved.

Y79 cells when treated with CA showed a dose dependent inhibition of cell proliferation. We observed 50% inhibition of Y79 cell proliferation at 50 μM concentration of CA. At the same concentration CA could only inhibit 20% proliferation of normal HCE cells. This indicates CA is more specific towards cancer cells. These differential effects of CA on cell proliferation between cancer and normal cells might be due to differences in constituent expression of the cellular protein signatures like constitutive NFκB expression [26].

Retinoblastoma cells grow in clusters, but with the CA treatment, the morphology and growth pattern of the cells changed considerably. Y79 cells with CA treatment showed distorted morphology, membrane blebs and less cell clusters. Nuclear and DNA fragmentation, a hallmark property of cells undergoing apoptosis, was observed in Y79 cells with CA treatment.

MMP is critical for proper cellular functions. Depolarized mitochondria lead to leakage of the membrane leading to release of Cytochrome *c* and formation of apoptosome complex [47]. Rhodamine 123 was used to assess the MMP in CA treated Y79 cells by flowcytometric analysis. Treatment with CA decreased the MMP of the retinoblastoma cells, indicated by the movement of cells towards lower scale on x axis of the dot plot. This disruption of MMP might alter the membrane dynamics of mitochondria leading to the molecular events triggering cell death. Similar disruption in membrane potential was observed with methanolic extracts of *Gracilaria tenuistipitata* in oral cancer cells [38, 48].

Bcl2 family members regulate the mitochondrial pathway of apoptosis. They are either pro apoptotic (Bak or Bax) or anti apoptotic (Bcl2 or Bcl XL). These proteins play role in permeabilization of the mitochondrial outer membrane on receiving apoptotic signals. Permeabilization leads to release of Cytochrome *c*, formation of apoptosome complex, activation of caspases, thus triggering morphological changes like membrane blebbing and nuclear fragmentation. Expression ratio of pro apoptotic BAX and anti apoptotic Bcl2 is the critical factor in driving cells towards life or death. Cells undergoing apoptosis show an increase in the expression of BAX and a decrease in the expression of Bcl2. 7α-hydroxy-β-sitosterol, a natural phytosterol oxide was shown to induce apoptosis by altering Bcl2/BAX ratio [49]. In our study, Y79 cells treated with CA shifted the ratio of Bcl2 and BAX towards cell death by increasing expression of BAX and decreasing the expression of Bcl2, release of cytochrome c and activation of caspase 3.

NFκB is a proliferating promoting protein and is activated in majority of tumors. NFκB translocates to the nucleus and activates transcription of several genes which are involved in cell proliferation. Bcl2 expression is defective in B cells that lack constituent proteins of NFκB indicating anti apoptotic Bcl2 might be regulated by NFκB [24]. The activation of NFκB is regulated by IκBα. We estimated the expression levels of NFκB and IκBα using western blotting. Treatment of retinoblastoma cells with CA inhibited the translocation of NFκB to the nucleus. CA suppressed the degradation of IκBα. This increase in levels of IκBα might have effected the translocation of NFκB to nucleus, effecting expression of several genes involved in Y79 cell proliferation. Similar NFκB mediated inhibition of cell proliferation was shown by natural products and plant extracts [50, 51].

**Figure 9:**
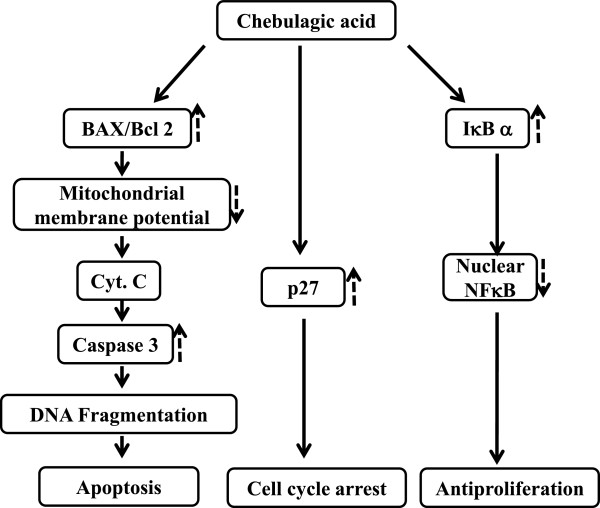
**Proposed schematic model for chebulagic acid mediated G1 arrest and apoptosis of Retinoblastoma cells Y79.**

Most of the anti cancer agents target the cell cycle of the cancer cells and this property to alter the cell cycle is considered one of the important property in the anti cancer drug development [52]. Progression of cell cycle is regulated by CDK inhibitor, p27. Increase in the expression of p27 inhibits the cell cycle entry into S phase [25]. Cell cycle analysis of the Y79 cells treated with CA showed an increase apoptotic or hypodiploid cells. We could also observe an increase in number of cells in G1 phase with CA treatment. Western blot analysis of p27 also showed a time dependent increase in expression, which indicates that CA induces G1 arrest by the induction of p27 expression. The present study thus demonstrates that CA induces apoptosis in retinoblastoma cell line Y79 by altering expression of Bcl2 family members, activating caspase 3, disrupting MMP and interfering with the NFκB cell proliferation pathway (Figure 9).

## Conclusions

Our findings clearly demonstrate that CA induces G1 arrest, inhibits NFκB and induces apoptosis of retinoblastoma Y79 cells. CA induced the release of Cytochrome *c* by modulating the MMP and altering BAX and Bcl2 ratio towards apoptosis. CA induced apoptosis of Y79 cells is caspase 3 dependent. G1 arrest in the Y79 cells with CA treatment is mediated by the increase in the expression of CDK inhibitor p27. CA inhibited the nuclear translocation of NFκB by suppressing the degradation of IκBα. This study highlights the potential of CA in inhibiting the proliferation of retinoblastoma cells. Further studies are required to assess the in vivo anti proliferative potential of CA. CA and any other analogs derived from it may be explored and validated further for its usefulness as anti cancer agents for retinoblastoma and other cancers.

## Abbreviations

CA: Chebulagic acid
CDK: Cyclin dependent kinase
COX: Cyclooxygenase
LOX: Lipoxygenase
FBS: Fetal bovine serum
DMEM: Dulbecco’s modified eagle’s medium
PBS: Phosphate buffered saline
MMP: Mitochondrial membrane potential.
